# RanGAP‐mediated nucleocytoplasmic transport of Prospero regulates neural stem cell lifespan in Drosophila larval central brain

**DOI:** 10.1111/acel.12854

**Published:** 2018-12-13

**Authors:** Di Wu, Litao Wu, Huanping An, Hongcun Bao, Pengfei Guo, Bei Zhang, Huimei Zheng, Fan Zhang, Wanzhong Ge, Yu Cai, Yongmei Xi, Xiaohang Yang

**Affiliations:** ^1^ Division of Human Reproduction and Developmental Genetics The Women's Hospital School of Medicine Zhejiang University Hangzhou China; ^2^ Institute of Genetics Zhejiang University Hangzhou China; ^3^ Department of Genetics, School of Medicine Zhejiang University Hangzhou China; ^4^ College of Life Sciences Zhejiang University Hangzhou China; ^5^ Temasek Life Sciences Laboratory National University of Singapore Singapore; ^6^ Department of Biological Sciences National University of Singapore Singapore; ^7^ Joint Institute of Genetics and Genomic Medicine between Zhejiang University and University of Toronto, Zhejiang University Hangzhou China; ^8^ Present address: Hanzhong Vocational and Technical College Hanzhong China; ^9^ Present address: Washington State University Spokane Washington

**Keywords:** cell cycle exit, *Drosophila* neuroblast, nucleocytoplasmic transport, Prospero, RanGAP

## Abstract

By the end of neurogenesis in *Drosophila *pupal brain neuroblasts (NBs), nuclear Prospero (Pros) triggers cell cycle exit and terminates NB lifespan. Here, we reveal that in larval brain NBs, an intrinsic mechanism facilitates import and export of Pros across the nuclear envelope via a Ran‐mediated nucleocytoplasmic transport system. In *rangap* mutants, the export of Pros from the nucleus to cytoplasm is impaired and the nucleocytoplasmic transport of Pros becomes one‐way traffic, causing an early accumulation of Pros in the nuclei of the larval central brain NBs. This nuclear Pros retention initiates NB cell cycle exit and leads to a premature decrease of total NB numbers. Our data indicate that RanGAP plays a crucial role in this intrinsic mechanism that controls NB lifespan during neurogenesis. Our study may provide insights into understanding the lifespan of neural stem cells during neurogenesis in other organisms.

## 1 INTRODUCTION

In *Drosophila*, neurogenesis occurs in two sequential steps. Neuroblasts (NBs), also known as neural stem cells, first form from the neuroectoderm in embryos and undergo a series of asymmetric divisions to produce self‐renewed NBs and ganglion mother cells (GMCs). The GMCs then divide terminally to generate neurons or glial cells to build the larval central nervous system (CNS). Once embryogenesis is completed, most abdominal NBs are eliminated through programmed cell death, whereas the cephalic and thoracic NBs enter mitotic quiescence at the embryo‐larval transition (Prokop & Technau, 1991; Tsuji, Hasegawa, & Isshiki, 2008). After the hatching of the larvae, most of the remaining NBs are reactivated and resume asymmetric divisions in the late first‐instar or early second‐instar larval stages to contribute ∼90% of cells in the adult CNS (Sousa‐Nunes, Cheng, & Gould, 2010; Srinivasan et al., 1998).

Prospero (Pros) is a homeodomain transcription factor expressed in NBs (Doe, Chulagraff, Wright, & Scott, 1991; Matsuzaki, Koizumi, Hama, Yoshioka, & Nabeshima, 1992; Ryter, Doe, & Matthews, 2002). Although it is a transcription factor, Pros only localizes to the basal cell cortex of mitotic NBs and is exclusively segregated into GMCs at telophase during NB asymmetric divisions (Hirata, Nakagoshi, Nabeshima, & Matsuzaki, 1995; Spana & Doe, 1995). In the newly formed GMCs, Pros detaches from the cell cortex and enters the nucleus where it exerts its function as a transcription factor (Hirata et al., 1995; IkeshimaKataoka, Skeath, Nabeshima, Doe, & Matsuzaki, 1997; Matsuzaki, Ohshiro, Ikeshima‐Kataoka, & Izumi, 1998; Srinivasan et al., 1998). The GMC with nuclear Pros then divides terminally and exits the cell cycle (Li & Vaessin, 2000).

By the white prepupal stage at the end of neurogenesis, the NBs begin to exit the cell cycle and cease proliferation. The characteristic feature for the termination of NBs, both in the brain and thoracic segments, is the translocation of Pros into the nucleus (Cenci & Gould, 2005; Maurange, Cheng, & Gould, 2008). Studies have shown that as Pros enters the nucleus, the thoracic NBs precede to a slow and final cell division to produce two equal‐sized daughters, resulting in NB cell cycle exit and the end of NB lifespan (Cenci & Gould, 2005; Maurange et al., 2008). The central brain NBs exit the cell cycle in a similar way (Chai, Liu, Chia, & Cai, 2013). By the end of neurogenesis at the pupal stage in *Drosophila*, all NBs, including those from the brain, thoracic, and abdominal segments, cease to have any proliferation potential. No actively dividing NBs have been found in adult flies (Fernandez‐Hernandez, Rhiner, & Moreno, 2013; Trotha, Egger, & Brand, 2009).

For a given NB, the time to exit the cell cycle is precisely regulated and in a fixed order. It has been proposed that the basic molecular mechanism regulating NB cell cycle exit involves a stereotypical temporal series of transcription factors being sequentially expressed in the NBs (Isshiki, Pearson, Holbrook, & Doe, 2001; Maurange et al., 2008; Rossi, Fernandes, & Desplan, 2017; Syed, Mark, & Doe, 2017; Tsuji et al., 2008). This temporal transcription factor series was first identified in the proliferating embryonic NBs in the ventral nerve cord (VNC), where a serial of transient expressed transcription factors, such as Hunchback (Hb), Krüppel (Kr), Pdm, Castor (Cas), and Grainyhead (Grh), took place according to the developmental stages of the embryo (Grosskortenhaus, Pearson, Marusich, & Doe, 2005; Isshiki et al., 2001). Under the control of temporal patterning regulation, Pros was transiently detected in the NB nucleus and NBs exited cell cycle, becoming quiescent at the end of embryonic neurogenesis (Lai & Doe, 2014; Li & Vaessin, 2000).

Temporal specification is not limited to embryogenesis but also occurs during postembryonic neurogenesis (Cenci & Gould, 2005; Chai et al., 2013; Maurange & Gould, 2005; Maurange et al., 2008). Two transcription factors, Cas and Seven‐up (Svp), have been reported to act as members of the postembryonic temporal series (Maurange et al., 2008; Syed et al., 2017). A recent study showed that the Hedgehog signaling pathway acts downstream of Cas but upstream of Grh to promote NB cell cycle exit (Chai et al., 2013). Other members in the temporal series for the postembryonic central brain remain to be identified.

Ran is a small Ras‐related GTPase that mediates the nucleocytoplasmic exchange of macromolecules across the nuclear envelope. Extensive studies in nuclear trafficking have shown that Ran acts as a molecular switch to regulate the assembly and disassembly of nuclear transport receptor–cargo complexes (Lui & Huang, 2009; Matchett et al., 2014).

RanGAP, a RanGTPase activating protein, acts to stimulate the hydrolysis of Ran‐bound GTP to GDP (Hutten, Flotho, Melchior, & Kehlenbach, 2008; Seewald, Korner, Wittinghofer, & Vetter, 2002). In mammalian cells, RanGAP is enriched in the cytoplasm and on the nuclear membranes (Zhang et al., 2015). In *Drosophila* primary spermatocytes and salivary gland cells, RanGAP is mainly localized at the outer periphery of the nuclear envelope (Kusano, Staber, & Ganetzky, 2001, 2002 ). In *Drosophila,* RanGAP is also highly expressed in many other tissues, including the CNS (https://flybase.org/reports/FBgn0003346.html). RanGEF is a chromatin‐bound guanine‐nucleotide exchange factor which converts RanGDP to RanGTP in the nucleus (Bischoff & Ponstingl, 1991). Human RanGEF is the regulator of chromosome condensation 1 (RCC1). *Drosophila* RanGEF is also known as Rcc1 or Bj1 (Joy, Hirono, & Doe, 2015). RanGAP and RanGEF (Rcc1) work together to maintain a functional Ran cycle. RanGTP in the cytoplasm is converted to RanGDP in the presence of RanGAP. RanGDP then enters the nucleus and exchanges GDP with GTP via Rcc1 function. RanGTP then moves out of the nucleus into the cytoplasm, completing a Ran cycle. This energy‐coupled Ran cycle, together with other proteins including Importins, Exportins, and nucleopore protein complex (NPC), is responsible for nucleocytoplasmic transport (Alavian, Politz, Lewandowski, Powers, & Pederson, 2004; Matchett et al., 2014; Nagai & Yoneda, 2012; Sampathkumar et al., 2013). Dysfunction of the Ran cycle disrupts the nucleocytoplasmic trafficking that involved in a number of neurodegenerative diseases (Freibaum et al., 2015; Zhang et al., 2015). A recent study has also suggested that Rcc1 acts to promote the nuclear export of Pros in the NBs and that a mutation in Rcc1 leads to premature NB differentiation in the central brain (Joy et al., 2015).

In this study, we reveal that RanGAP is involved in an intrinsic mechanism that controls Pros nuclear translocation in larval central brain NBs. Mutations in *rangap* cause abnormal accumulation of Pros in the nuclei of NBs. This nuclear Pros leads to the cell cycle exit of NBs and the end of NB proliferating potential. Our data show that in the third‐instar larval central brain NBs, Pros shuttles across the nuclear envelope via a nucleocytoplasmic transport system. Elimination of the RanGAP function does not affect the import of Pros but the export of Pros out of the nucleus. RanGAP or the Ran‐mediated nucleocytoplasmic transport system may serve as an end target for the temporal series of transcription factor cascade in controlling the NB lifespan.

## 2 RESULTS

### 2.1 RanGAP is required for maintenance of NB number in the larval central brain

**Figure 1:**
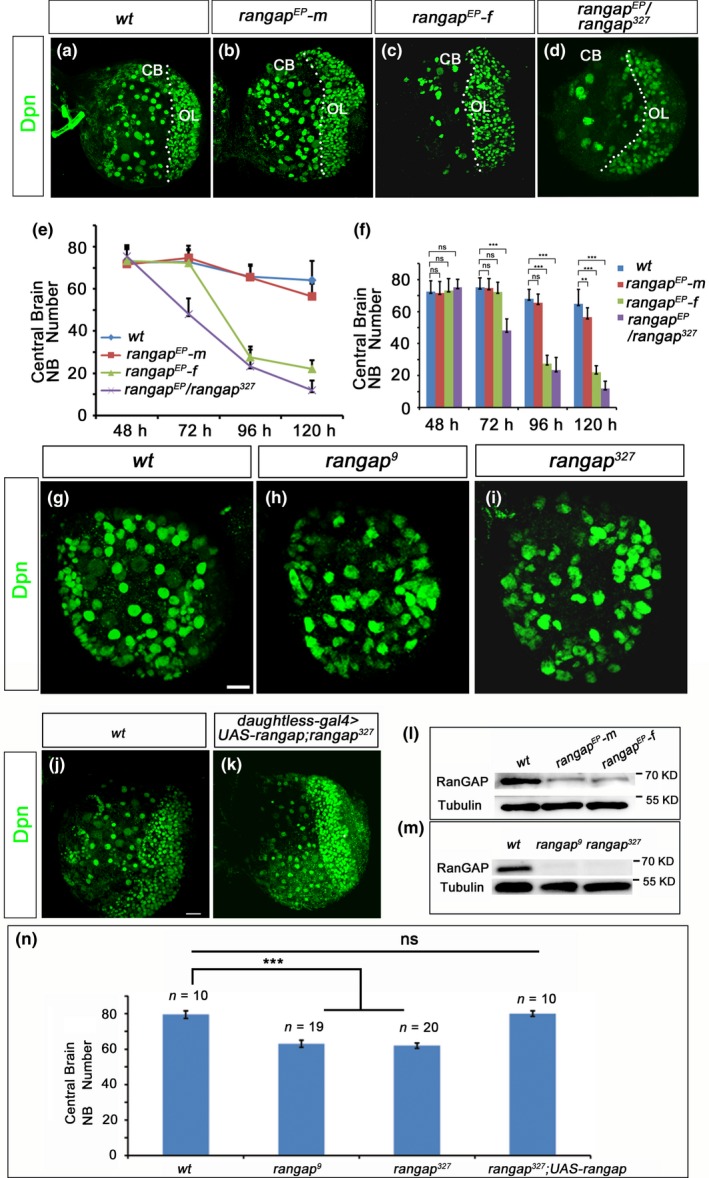
RanGAP is required for maintenance of NB numbers. (a–d) Confocal images from the projection of Z‐stack of the late third‐instar larval brains (96 hr ALH) of *wt *(a), *rangap^[EP1173]^* male (*rangap^EP^‐m*; b), and *rangap^[EP1173]^* female (*rangap^EP^‐f*; c) animals, and the trans‐heterozygous (*rangap^[EP1173]^*/*rangap^327^*; d). Brain lobes were immunostained with anti‐Dpn (green) to visualize the NBs in the central brain (CB). Compared with *wt* and *rangap^[EP1173]^*males, the NB numbers in *rangap^[EP1173]^*female and *rangap^[EP1173]^*/*rangap^327 ^*brains had decreased significantly. (e, f) Statistical analysis of the NB numbers/brain lobe at different developmental stages (48, 72, 96, 120 hr ALH) in the *wt* (*n* = 6, *n* = 8, *n* = 8, *n* = 10, respectively) and *rangap^[EP1173]^* brain lobes (male: *n* = 10, *n* = 10, *n* = 10, *n* = 15; female: *n* = 35, *n* = 40, *n* = 35, *n* = 45) and *rangap^[EP1173]^*/*rangap^327 ^*(*n* = 9, *n* = 9, *n* = 6, *n* = 11). According to the one‐way ANOVA test and Dunnett’s multiple comparison test, at 48 and 72 hr ALH, the NB numbers/lobe of both male and female animals were comparable to those of the *wt* brains.(at 48 hr, *p* = 0.6527 and 0.9683, at 72 hr, *p* = 0.0938 and 0.9637). The NB numbers of *rangap^[EP1173]^* female animals dropped significantly during the next 24 hr (*p* < 0.0001), for male, *p* = 0.6995. At 120 h ALH, NB numbers of *rangap^[EP1173]^* of both male and female are decreased (female, *p* < 0.0001; male, *p* = 0.0004). For trans‐heterozygous *rangap^[EP1173]^*/*rangap^327^* animals, NB numbers decreased at 72 hr ALH (*p* < 0.0001). (l) The western blot analysis of the brain extracts from the *wt* and *rangap^[EP1173]^*males and females. The RanGAP expression levels decreased in the *rangap^[EP1173]^*mutants*.* (g–i)The second‐instar larval brains of the *wt *(f), *rangap* ^9^ (g), and *rangap* ^327^ (h) groups were immunostained with Dpn (green). Both *rangap* ^9^ and *rangap* ^327^ NB number had decreased (l) as compared with the *wt*. (j, k) The confocal images of third‐instar larval brains of the *wt *(i) and *daughtless‐Gal4>UAS‐rangap;rangap^327^* were double‐labeled with anti‐Mira (red) and anti‐Dpn (green). Ectopic expression of RanGAP in *rangap^327^* background (*daughtless‐Gal4>UAS‐rangap; rangap^327^*) rescued the loss of NB number phenotype. (m) Western blot data showing that RanGAP was not detected in *rangap* ^9^ and *rangap* ^327^ extracts, and these two mutants were null alleles. (n) NB number comparisons among the *wt*, *rangap* ^9^, *rangap* ^327^ and the rescue with the ectopic expression of RanGAP (*daughtless‐Gal4>UAS‐rangap;rangap^327^*) at 48 hr ALH (2nd instar). Significant differences between *wt *and *rangap *mutants were tested using one‐way ANOVA test and Dunnett's multiple comparison test (*p* < 0.001). No significance in difference was detected between *wt *and *UAS‐rangap; rangap^327^* using the same test. CB, central brain; OL, optic lobe. White dashed lines define the border between the CB and OL. Scale bar = 10 μm

In an RNAi screen to identify the genes required for NB development, we observed the occasional mislocalization of Mira in the *rangap* RNAi knockdown larval central brain NBs (Supporting Information Figure S1A,B). This phenotype was confirmed in the third‐instar larval central brains of *rangap^[EP1173]^*, a mutation with a P‐element insertion in the 5′‐UTR region of the gene (Supporting Information Figure S1C,G). All *rangap^[EP1173]^* females died at the pupal stage but some male animals of the same genotype survived to the adulthood. Based on viability results, we focused on mutant female animals. We performed immunofluorescence staining at the third‐instar larval stage and found the gloss physical appearance of the brain lobes appeared to be normal (Figure 1a–c), but interestingly the mutant female animals had less total NB number as compared with *wt* (Figure 1a–c). One potential reason for this lower NB number in female brains could be the defective NB activation during late first‐ or early second‐instar larval stages (Maurange & Gould, 2005; Maurange et al., 2008; Sousa‐Nunes, Yee, & Gould, 2011). To explore this possibility, we quantified NB numbers of larval central brains at different developmental stages (Figure 1e,f). The total NB numbers in the *rangap^[EP1173]^* central brains were comparable to those of the *wt* at the second‐ (48 hr ALH) and early third‐instar larval stages (72 hr ALH), but dropped significantly to approximately 30% of the controls within the subsequent 24 hr. It is interesting to note that at a later stage (120 hr ALH), the NB numbers in male animals also drop notably (Figure 1e,f). This observation indicates that the reactivation of NBs is normal in the *rangap^[EP1173]^* female brains, and the disappearance of 70% of total NBs occurs in the late third‐instar larval stage.

To exclude the possibility that the NB missing phenotype initially identified in *rangap^[EP1173]^*allele was due to the background mutations, we examined *rangap^[EP1173]^*/*rangap* ^327^ trans‐heterozygous animals. Interestingly, trans‐heterozygous animals of both sexes were lethal at pupal stage and showed central brain NB missing phenotype (Figure 1d). The quantification of NB numbers in this genetic background showed that the central brain NB numbers were comparable to those of *wt* and other two null alleles at 48 hr (ALH) but started decreasing significantly at 72 hr (ALH). The NB numbers further dropped to <30% at 96 hr and <20% at 120 hr (ALH), respectively (Figure 1e,f). The stronger phenotype observed in trans‐heterozygous animals can be explained by a lower RanGAP expression levels (50% of that of *rangap^[EP1173]^* homozygous) and supports the proposal that NB missing phenotype exhibited in *rangap^[EP1173]^*allele is not due to background mutations.

We also suspected that apoptosis was the cause for the missing NBs, but the anti‐Caspase 3 immunofluorescence staining failed to detect any obvious apoptotic central brain NBs in the *rangap^[EP1173]^*female animal brains. The apoptotic signals in *rangap^[EP1173]^*female brains seemed to be identical to those of the *wt *(Supporting Information Figure S1H,I). Based on this observation, we conclude that the loss of NB number is unlikely due to the apoptosis.

Since *rangap^[EP1173]^*is a hypomorphic allele and shows NB loss only in female animals, it would be interesting to know whether the phenotype is linked to RanGAP expression levels. An antibody against a RanGAP fragment from aa280 to 426 was generated in rabbits and exhibited specificity against RanGAP protein. Bands with appropriate molecular weights for the endogenous RanGAP and a tagged RanGAP protein were detected, respectively, on western blot (Supporting Information Figure S1J). Western blot data further showed that the RanGAP expression levels in *rangap^[EP1173]^*mutant brains of both male and female animals were significantly decreased as compared with the *wt *(Figure 1l). However, we failed to detect any expression discrepancy between male and female brains (Figure 1l).

We generated two additional *rangap* alleles: *rangap* ^9^, a frame‐shift mutation with five bases deleted near the N‐terminal region with the CRISPR/Cas9 method and *rangap* ^327^, a nonsense mutation due to a G to T change (“GAG” to “TAG”) generated during remobilization of the P‐element from *rangap* ^[EP1173]^ (refer to Materials and Methods; Supporting Information Figure S1G). The homozygous animals of *rangap* ^9^ and *rangap* ^327^ mutants only survived up to the second‐instar larval stage. Western blot analysis showed that the RanGAP protein was not detectable for both alleles (Figure 1m). Immunofluorescent staining also failed to identify RanGAP signals in mutant NBs (Supporting Information Figure S1e,f). These data confirm that both *rangap* ^9^ and *rangap* ^327^ mutants are null alleles. We next quantified the central brain NB numbers of the second‐instar larvae in both null alleles. The total NB numbers in these mutants were only about 80% of those in *wt* counterparts regardless of the sex of the animals (Figure 1g,h,i).

The phenotypic differences between *rangap^[EP1173]^*male and female animals observed earlier must be due to the low expression levels of RanGAP and unknown complexed sex‐specific regulations. We decided to pursue only the relationship between RanGAP and the lifespan of the NBs.

The overexpression of a *rangap* transgene construct (RanGAP‐TAP) in the *rangap* ^327^ mutant background with daughterless‐Gal4 (Supporting Information Figure S2A) effectively rescued the missing NB phenotype (Figure 1j,k,n). Based on all these results, we conclude that RanGAP is required for the maintenance of NB numbers in larval central brains.

### 2.2 Lack of RanGAP causes nuclear Pros‐dependent NB cell cycle exit

**Figure 2:**
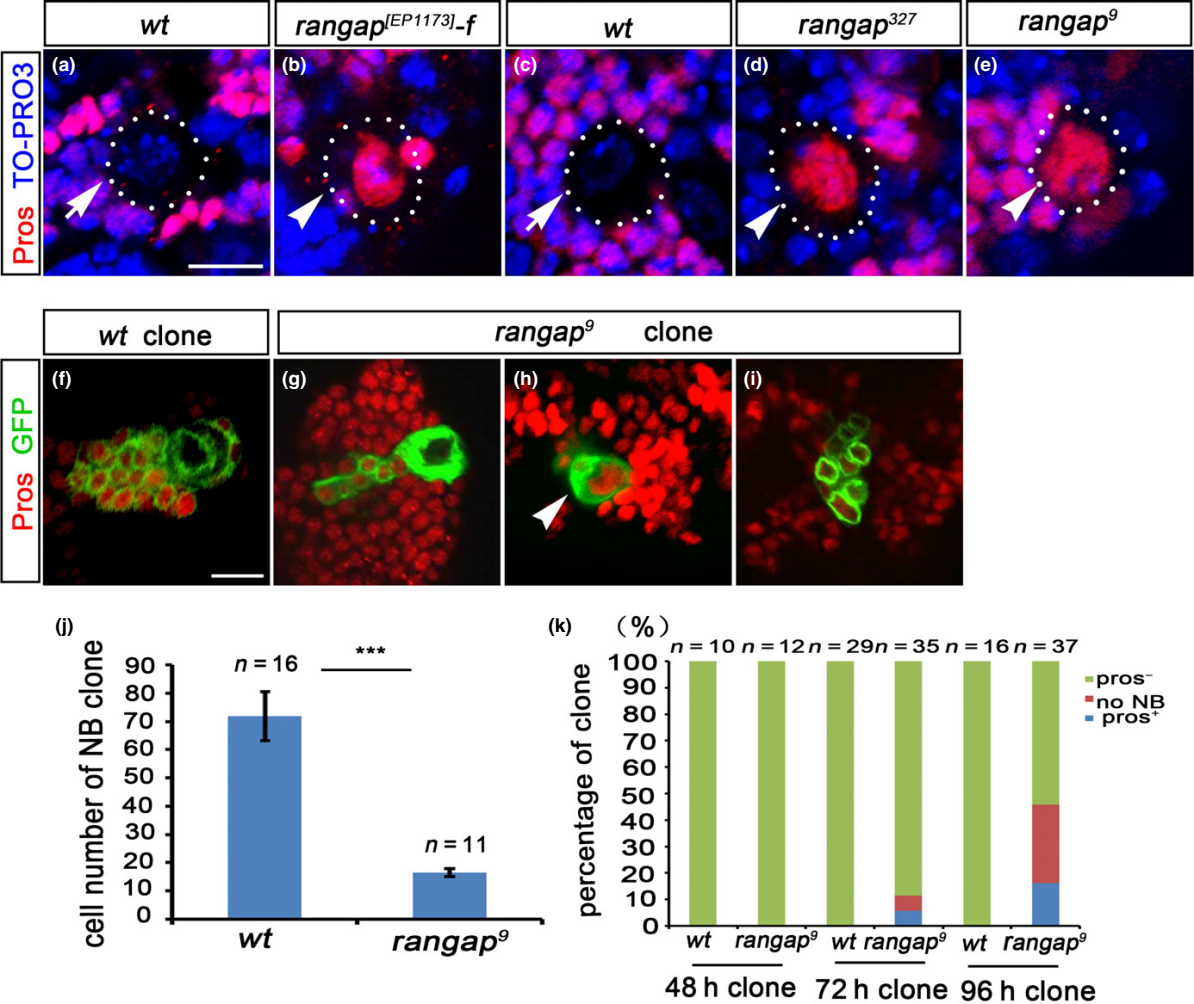
Lack of RanGAP leads to premature nuclear Pros‐dependent NB cell cycle exit. (a–e) Confocal images of the third‐instar larval brains of *wt *(a) and *rangap^[EP1173]^* female (b); and the second‐instar larval brains of the *wt *(c), *rangap^327 ^*(d), and *rangap^9^* (e) labeled by anti‐Pros (red) and TO‐PRO3 (blue). In *wt* central brain NBs, no nuclear Pros was detected (6 third‐instar brain lobes, 8 second‐instar brain lobes). The nuclear Pros (arrowhead) was detected in every single brain lobe of all three *rangap* mutants (*rangap^[EP1173]^*:10 brain lobes, *rangap^327^*:14 brain lobes, and *rangap^9^*: 11 brain lobes). On average, two to three NBs with nuclear Pros were detected in each lobe. (f–i) The third‐instar larval brains containing the *wt* (f), *rangap* ^9^ (g–i) MARCM clones double‐labeled by anti‐GFP (green) and anti‐Pros (red). The *wt* clones showed large NBs with many small cells (f) and no nuclear Pros detection in the NBs. The *rangap* ^9^ clones showed three distinct phenotypes: large NB with many small cells but no nuclear Pros in the NB (g); large NB with many small cells and nuclear Pros in the NB (arrowhead, h) and only small cells without NBs (i). (j) Statistical data of NB clone size (cell numbers/clone) of the *wt *and *rangap^9^* clones. The data are plotted as mean ± *SEM*. Significant differences were assessed using a two‐tailed unpaired *t* test (***p < 0.001). (k) The percentage of the three types of *rangap* ^9^ clones (with or without nuclear Pros, and clones without large NBs) at various developmental stages of 48, 72, and 96 hr ALH. Data information: Dotted lines mark the outline of the NBs. Scale bar = 10 μm

We suspected that the loss of NBs in *rangap* mutants was due to the nuclear Pros‐dependent NB cell cycle exit. As predicted, nuclear Pros was detected in the central brain NBs of the *rangap^[EP1173]^*, as well as in the two null allele mutants (Figure 2a–e, Supporting Information Figure S2B,C). In the *rangap^[EP1173]^* mutant female brains, nuclear Pros was found after 72 hr ALH (Figure 2b), and for *rangap* null alleles, nuclear Pros was observed in the second‐instar larval central brains (Figure 2d,e).

To further verify that NBs with nuclear Pros were able to exit the cell cycle and terminate their NB cell fate, we performed MARCM studies on the *rangap^9^* mutant (Wu & Luo, 2006). Based on the phenotypes, the MARCM clones were subdivided into three groups: Group 1: large NBs with many small cells and no nuclear Pros in the large cell (Figure 2g); Group 2: large NB with nuclear Pros and many small cells (Figure 2h); and Group 3: small cells only with no large NB (Figure 2i). Our interpretation is that these three groups reflect different stages of the nuclear Pros‐dependent NB cell cycle exit in the absence of RanGAP. Among *rangap* clones, Group 1 represented mutant NBs prior to the cell cycle exit; Group 2 showed mutant NBs in the process of cell cycle exit with nuclear Pros; and Group 3 showed the *rangap* clones post‐NB cell cycle exit. The ratio of these three groups varied with time. At 48 hr (ALH), only Group 1 clones were detected. At 72 hr (ALH), a few NB clones belonged to the Groups 2 and 3, while at 96 hr (ALH), about 40% of total NBs in the *rangap* clones had exited the cell cycle or were in the process of cell cycle exit (Groups 2 and 3; Figure 2k). We also examined the double mutant for RanGAP and Pros by using both RNAi lines and observed tumor‐like phenotype in the third‐instar larval central brain (Supporting Information Figure S2E–H).

The MARCM clone data also indicate that in the absence of RanGAP, the nuclear Pros‐dependent NB cell cycle exit starts from 72 hr (ALH). Prior to this time point, RanGAP might not be necessary for preventing nuclear Pros in the NBs. This conclusion is consistent with our previous observations of the NB loss in *rangap^[EP1173]^*.

### 2.3 RanGAP modulates NB cell cycle progression

**Figure 3:**
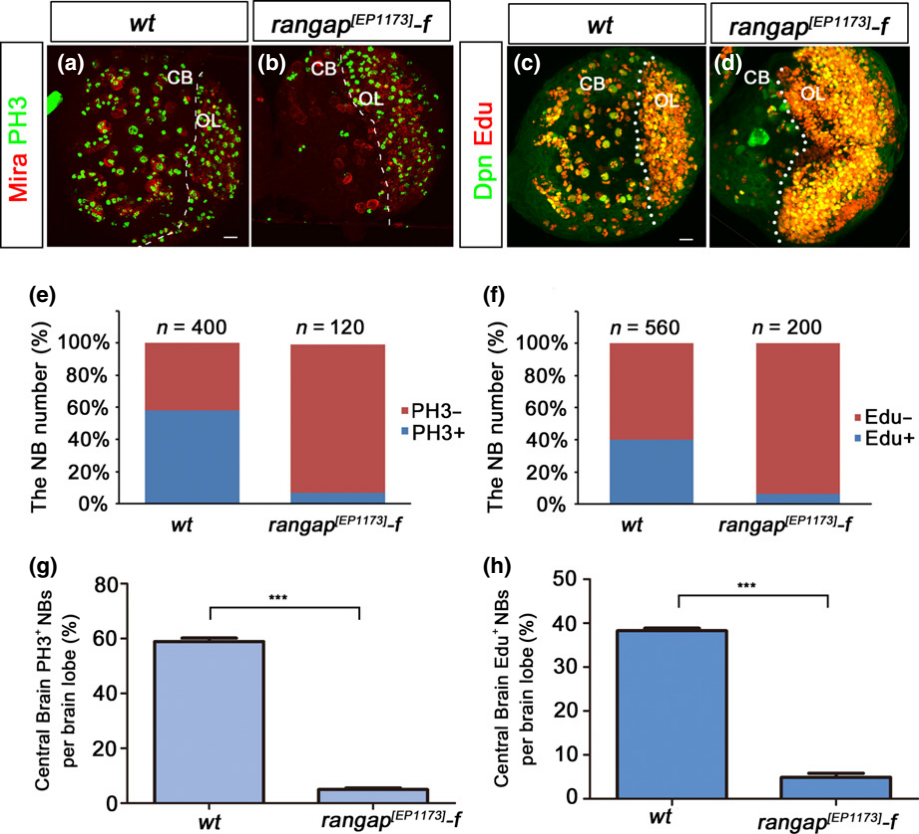
RanGAP facilitates NB cell cycle progression. (a, b) The confocal images of the third‐instar larval brains’ ventral view of the *wt *(a), *rangap^[EP1173]^*female (b) were double‐labeled with Mira (red) and PH3 (green) to show the NBs in the mitotic phase. (c, d) The third‐instar larval brains of *wt *(c) and *rangap^[EP1173]^‐f *(d) are stained with Edu (red) to show the NBs in S‐phase. (e) The PH3^+^ NBs in *wt* were about 58% in the total NB population while only 7% in the *rangap^[EP1173]^‐f*. From the two‐tailed unpaired *t* test, the PH3^+^ NBs in the central brain of *rangap^[EP1173]^‐f* are much less than *wt *(*p* < 0.0001). And the confidence interval for *wt *and *rangap^[EP1173]^‐f* is 0.5595–0.6190 and 0.03186–0.06687, respectively. (f) The Edu^+^ NBs in *wt* were about 40% in the total NB population while only 6% in the *rangap^[EP1173]^‐f*. According to the two‐tailed unpaired *t* test, there was big decrease of Edu^+^ NBs in rangap p‐line (*p* < 0.0001). CB: central brain; OL: optic lobe. White dashed lines define the border between the CB and OL. Scale bar = 10 μm. (g) Detailed analysis of the PH3^+^ NBs difference between *wt* and *rangap^[EP1173]^‐f *from figure e (*p* < 0.0001). The data are plotted as mean ± *SEM*. In these two groups, we analyzed seven and five brain lobes, respectively. (h) Detailed analysis of Edu^+^ NBs per brain lobe in wt and *rangap^[EP1173]^*‐f from figure f (*p* < 0.0001). Ten and eight brain lobes were analyzed, respectively. The error bars show the variation of the percentage. The data are plotted as mean ± *SEM*

We noticed that in our MARCM clone analyses, the Group 1 NBs appeared to be less affected by the loss of RanGAP. One obvious feature that made NBs in Group 1 different from the *wt* counterparts was the smaller clone size (Figure 2j). This suggested that in the *rangap* mutant, the NB cell cycle was either delayed or stalled. To address this issue, we carried out Edu and PH3 labeling experiments to monitor S‐phase and M‐phase NBs. At 96 hr (ALH) in *rangap^[EP1173]^* female brains, the total numbers of NBs labeled by PH3 (Figure 3a,b) or Edu (Figure 3c,d) were much less than those of the *wt*. Statistical analysis showed that only about 7% of NB populations were in the mitotic phase and 6% in or post‐S‐phase, while the percentages for the *wt *counterparts were 58% and 40%, respectively (Figure 3e‐h). Based on these data, we conclude that RanGAP is required for correct NB cell cycle progression. This cell cycle delay or halt might be a result of the nuclear Pros‐dependent cell cycle exit.

### 2.4 Cytoplasmic RanGAP negatively correlates with nuclear Pros

**Figure 4:**
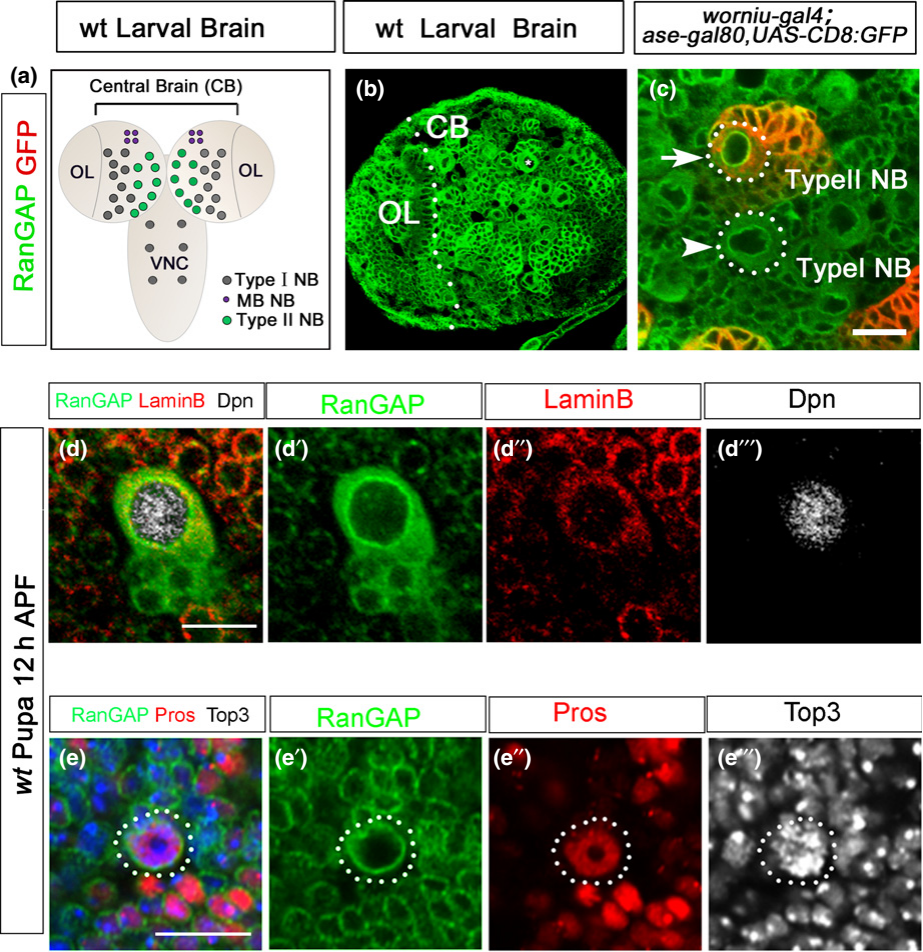
Cytoplasmic RanGAP is negatively correlated with nuclear Pros. (a) The diagram of a *wt* third‐instar larval brain showing the Type I and Type II NBs in the central brain. Adopted from Homem et al (2015 *Nat. Rev. Neurosci* 16(11):647–659). (b, c) The confocal images of the third‐instar larval brains stained with anti‐RanGAP (green, b) and double‐labeled with anti‐RanGAP (green) and GFP (red) to show that RanGAP is expressed in both Type I (arrowhead) and Type II (red, arrow) NBs (c). The fly line *wor‐Gal4,ase‐Gal80,UAS‐CD8::GFP* marks the Type II NBs (red). (d) The confocal images of *wt *brains at 12 hr after pupa formation (12 hr APF)triple labeled with anti‐RanGAP (green), Lamin B (red), and Dpn (white). The NB without nuclear Pros showed clear RanGAP expression both in the cytoplasm and on the nuclear envelope (d–d′′′). (e) The confocal images of *wt *brains at same stage triple labeled with anti‐RanGAP (green), Pros (red), and TO‐PRO3 (white). In the NBs with nuclear Pros, the cytoplasmic RanGAP had disappeared, and RanGAP was only visible on the nuclear envelope (100%, *n* = 16, E–E′′′). The white dotted line defines the border between the CB and the OL. White dotted circles mark the outline of the NBs. Scale bar = 10 μm

RanGAP was widely expressed in the NBs and other cells in both the central brain (CB) and the optic lobes (OL; Figure 4a,b). We focused on the central brain NBs. At interphase, RanGAP was in the cytoplasm and restricted to the nuclear envelope in both Type I and Type II NBs (arrowhead and arrow, Figure 4c). This subcellular distribution pattern is reminiscent of RanGAP localization in mammalian cells. Studies on mammalian systems suggest that RanGAP existed in two states in cells: the soluble form in the cytoplasm and the SUMOlysated form with nuclear pore complex (NPC) through Nup358 (RanBP2; Hutten et al., 2008; Mahajan, Delphin, Guan, Gerace, & Melchior, 1997). Since RanGAP is highly conserved between the mammals and *Drosophila*, it is likely that *Drosophila* RanGAP also has these two forms.

In order to distinguish the potential functions of cytoplasmic RanGAP from the nuclear membrane‐bound SUMOylated one, we examined the relationship between RanGAP distribution and nuclear Pros in the NB cell cycle exit. At 12 hr after pupa formation (APF), a closer look at the RanGAP subcellular distribution revealed that in the control NBs without nuclear Pros, RanGAP was clearly detected both in the cytoplasm and on the nuclear envelope (Figure 4d–d′′′). But in NBs with nuclear Pros, the cytoplasmic RanGAP had disappeared and only the nuclear envelope bound RanGAP was visible (Figure 4e‐e′′′). This observation suggests that cytoplasmic and nuclear membrane‐bound RanGAP may have different functions. The cytoplasmic RanGAP levels negatively correlate with the nuclear Pros in the NBs. The RanGAP expression becomes more restricted in pupal brains. Total 16 NBs with nuclear Pros were observed in pupal brains, and none of them showed cytoplasmic RanGAP distribution. This result is consistent with the report in mammalian cells that soluble RanGAP is involved in a basic disassembly mechanism which is responsible for the disassociation of both trimeric export complexes and the recycling import/RanGTP complexes in nucleocytoplasmic transport (Ritterhoff et al., 2016).

### 2.5 RanGAP prevents nuclear Pros retention via the Ran cycle

**Figure 5:**
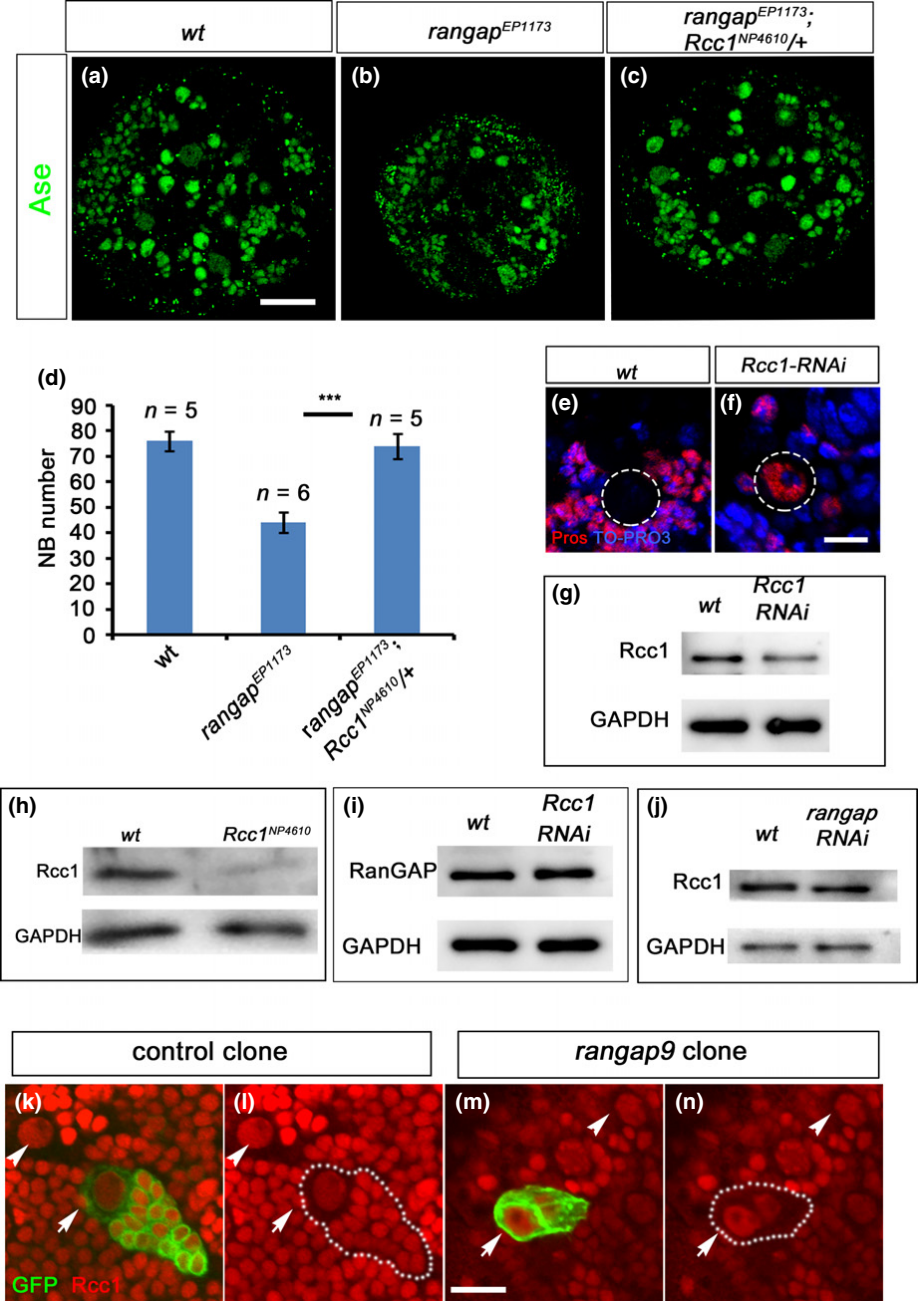
Premature appearance of nuclear Pros is due to a defective Ran‐mediated nucleocytoplasmic transport system. (a–c, h) The confocal images of NBs of the third‐instar larval brains stained with anti‐Ase (green). The loss of NB number phenotype in *rangap^EP1173^* mutant brains (b) was rescued by introducing one copy of the hypomorphic mutant *Rcc1^NP4610^* (h) into the *rangap^EP1173^* mutant background (c). (d) Statistic analysis of rescue results. From the one‐way ANOVA test and Dunnett's multiple comparison test, removal of one copy of RCC1 in *rangap^EP1173^*, the NB number is greatly restored (*p* < 0.0001). *n*: number of the brain lobes examined. Scale bar = 20 µm. (e–f) The knockdown of *Rcc1* with worniu‐Gal4 led to a premature nuclear Pros phenotype. Dotted circles outline NB shape. Scale bar = 10 µm. (g, i–n) Expression of Rcc1 or RanGAP was not mutually dependent. The western blot data show that neither *Rcc1 *knockdown (g) in the third‐instar larval brain NBs affect RanGAP expression (g, i) nor the attenuation of *rangap* expression alter Rcc1 expression (j). In addition, the anti‐Rcc1 signal (red) remained unchanged both in the control clone (k, l) and *rangap^9^* MARCM clones (arrow; *n* = 14, m, n), comparing with its neighboring *wt* NB (arrowhead). GFP (green). The dotted line outlines the clone size

It is known that RanGAP and Rcc1 work together to balance the RanGTP/RanGDP ratio in the Ran cycle (Nagai & Yoneda, 2012). When RanGAP activity is low, RanGTP will accumulate in the cytoplasm and the RanGTP/RanGDP ratio is altered. Reduction of Rcc1 expression should be able to compensate for the lowered RanGAP activity, reset the RanGTP/RanGDP ratio, and rescue the *rangap* phenotype. By introducing a copy of the *Rcc1^NP4610^* mutant gene to attenuate Rcc1 levels (Figure 5h) in *rangap^[EP1173]^*mutant female animals (Figure 5b), we observed the restoration of NB numbers (Figure 5c,d) and the animals survived to adulthood. In addition, we did not observe any NBs (*n* = 368) with nuclear Pros in the third‐instar larval central brains of these animals. This result indicates that the retention of nuclear Pros in the *rangap* mutant NBs is due to a defective Ran cycle.

A recent study has showed that the knockdown of *Rcc1* in larval central brain NBs led to premature nuclear Pros phenotype identical to that of the *rangap* mutants (Figure 5e,f; Joy et al., 2015). This prompted us to explore the potential relationship between RanGAP and Rcc1. To exclude the possibility that these two proteins were mutually regulated, we proceeded to evaluate the expression levels of RanGAP and Rcc1 in the reciprocal mutant brains. Our western blot data showed RanGAP expression (Figure 5i and Supporting Information Figure S2D) in the *Rcc1 *RNAi treated brain extracts or Rcc1 expression in the brains treated with *rangap* RNAi had remained unchanged (Figure 5j and Supporting Information Figure S2D). Furthermore, it was clear that Rcc1 expression in *rangap^9^* NB MARCM clones remained unchanged (Figure 5k–n). This experimental result indicates that the expression of RanGAP or Rcc1 is independent. It has been reported that RanGAP function is associated with protein import into the nucleus and that Rcc1 associates with protein export (Fujiwara, Hasegawa, Oka, Yoneda, & Yoshikawa, 2016; Joy et al., 2015; Ritterhoff et al., 2016; Zhang et al., 2015).

This appeared contradictory to the observation that mutations in either *rangap* or *Rcc1* resulted in an identical premature nuclear Pros phenotype since RanGAP and Rcc1 had opposite roles upon Ran status. It is conceivable that in the nucleocytoplasmic transport system, energy coupled to maintain the transport system comes from the hydrolysis of GTP in the Ran cycle. Mutations in *rangap* or *Rcc1* would then ultimately lead to malfunction in the Ran cycle and a defective nucleocytoplasmic transport system in the NBs. In this scenario, it is possible that the appearance of premature nuclear Pros in both mutants was due to a defective Ran‐mediated nucleocytoplasmic transport system.

### 2.6 Pros shuttles across nuclear envelope in NBs

**Figure 6:**
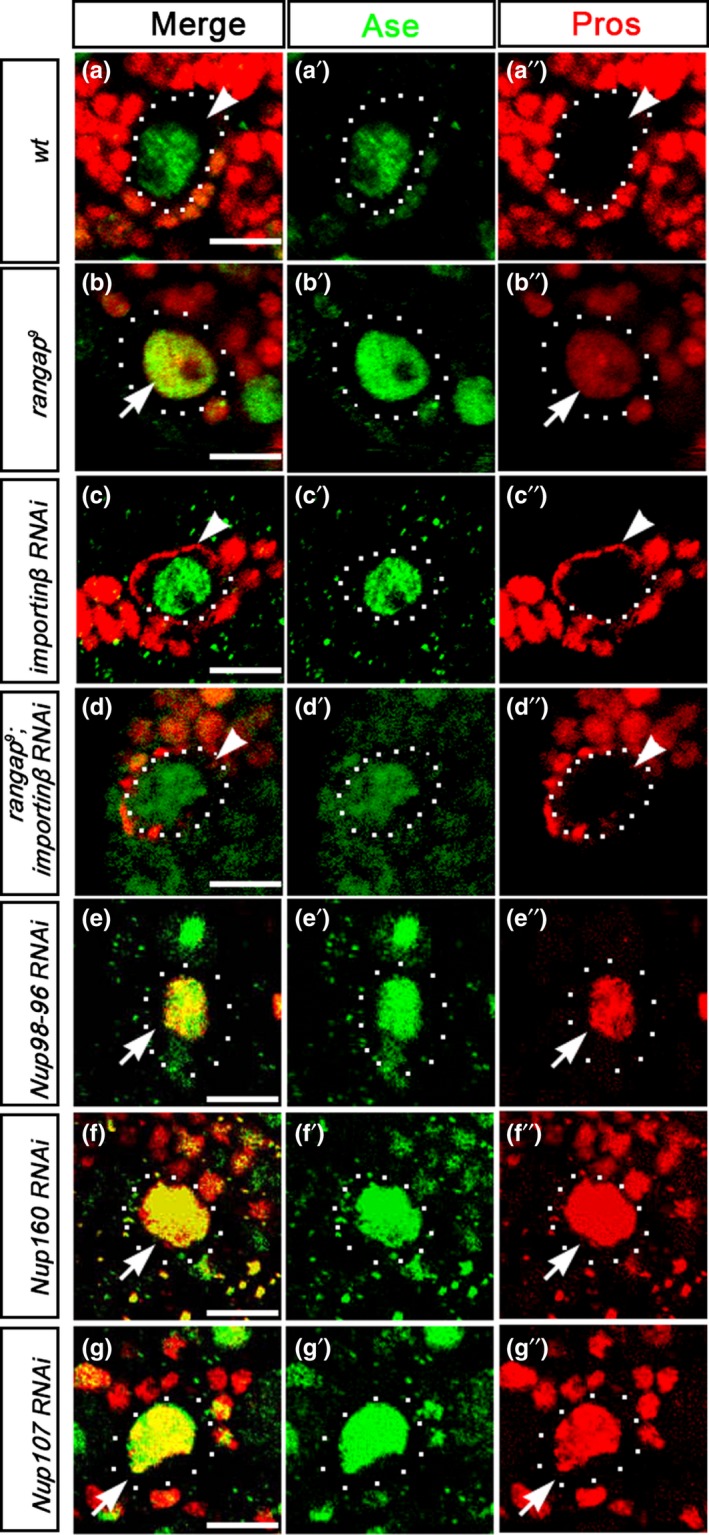
Transcription factor Pros is constantly shuttling across the nuclear envelope in *wt* NBs. (a–a′′) The localization pattern of Pros in *wt* third‐instar larval brains. Pros is not nuclear or cortical in the NBs. (brain lobes = 15). (b–b′′) In the absence of RanGAP, premature nuclear Pros accumulation was detected in the NBs (arrow) in the second‐instar larval brains of *rangap^9^*. Nuclear Pros was detected in two or three NBs per lobe (brain lobes = 21). This low number agrees with our finding that at 48 hr ALH, few NBs contain nuclear Pros (refer to Figure 2k).(`c–c′′) Attenuation of Importin β expression in the third‐instar larval brains led to cortical Pros in 140 NBs (arrowhead) from 14 lobes observed. (d–d′′) When *importin β* was knocked down in the *rangap* mutant background (arrowhead), the barely visible anti‐Pros staining was detected in 287 NBs from 11 second‐instar larval brain lobes. This Pros distribution was reminiscent of the pattern seen in the *wt* NBs (a–a′′). (e–g′′) Attenuated expression of three NPC proteins, Nup98‐96, Nup160, or Nup107, led to nuclear Pros in one to two NBs per lobe (arrow) from 10, 7, and 9 third‐instar larval brain lobes, respectively. In wt third‐instar larval brain lobes, nuclear Pros is never observed in NBs. Dotted circles outline the NBs. Scale bar = 10 µm

Pros has both a nuclear localization signal (NLS) and a nuclear export signal (NES) which implies that Pros is able to be imported into and exported out of the nucleus (Demidenko, Badenhorst, Jones, Bi, & Mortin, 2001; Vaessin et al., 1991). To elucidate the mechanism of how Pros accumulates in the nucleus in the absence of RanGAP (Figure 6a,b), we investigated whether the Importins play a role in Pros nuclear translocation. In the cytoplasm, the carrier protein Importin β forms complex with Importin α and the cargo proteins for the first step of protein import (Hutten et al., 2008). When Importin β was attenuated using RNAi in the interphase larval central brain NBs, Pros was largely cortical (arrowhead, Figure 6c), while Pros staining in *wt* NBs was quite weak, sometimes barely visible in the cytoplasm (arrowhead, Figure 6a).

This result suggests that Importin β is involved in the import of Pros into the nucleus. If Importin β was not participating in the import of Pros into the nucleus, knockdown of Importin β should have not affected Pros localization in the NBs. Since Pros is not detected in the nucleus in *wt* NBs, imported Pros must be exported back to cytoplasm at the same rate so that Pros does not accumulate in the nucleus and remains mainly in the cytoplasm. This observation strongly suggests that Pros is constantly imported into and exported out of the nucleus, shuttling across the nuclear envelope in *wt* NBs.

One of the conceivable explanations why attenuation of Importin β levels in the NBs led to a cortical Pros distribution could be simply the block of Pros import into the nucleus. It is likely that in interphase NBs, both cytoplasm and nucleus contain certain basal levels of Pros which are not easily visualized by antibody staining. As Pros shuttles across the nuclear envelope, knockdown of Importin β expression only halts the import of Pros, and the export of Pros is still functional. Under this circumstance, the majority of Pros, including that from the nucleus, is enriched in the cytoplasm resulting in the cortical visualization of Pros in the NBs.

### 2.7 Pros export is defective in the absence of RanGAP

The observation that, in the absence of RanGAP, Pros is detected in the nucleus suggests that the import of Pros remains unaffected, while the export of Pros is compromised in mutant NBs. To verify this hypothesis, we carried out genetic manipulations to remove both Importin β and RanGAP in the larval brain NBs. We reasoned that in this import and export double‐blocking experiment, Pros should remain in its original compartments, and the nuclear accumulation of Pros should not occur. When Pros import/export was blocked by the attenuation of Importin β in the *rangap^9^* mutant background, nuclear Pros accumulation was not detected (arrowhead, Figure 6d) and the Pros immunofluorescence signal remained weak and comparable to that of *wt* NBs (arrowhead, Figure 6a). This result is consistent with our Pros shuttling hypothesis.

We next searched for other components involved in the protein export in nucleocytoplasmic transport system. Three nuclear pore proteins, Nup 107, Nup 160, and Nup 98‐96, were of particular interest. It has been reported that mutations in these three proteins suppress G4C2 repeats‐mediated neurotoxicity toxicity in the *Drosophilar* eyes (Freibaum et al., 2015). The mechanism of the suppression was most likely due to decreased protein export since KPT‐276, a small‐molecule inhibitor of nuclear export, also eased this toxicity (Zhang et al., 2015). Thus, these three nuclear pore proteins are likely involved in protein export.

When *Nup 107*, *Nup 160,* or *Nup 98‐96* was knocked down individually, a premature retention of nuclear Pros was observed (Figure 6e–g). Attenuation of Nup 107, Nup 160, or Nup 98‐96 expression levels diminishes protein export capacity and recapitulates the *rangap* mutant phenotype. Based on these results, we conclude that the attenuation of nuclear export is able to cause premature accumulation of Pros in the nucleus. This conclusion is consistent with our hypothesis that *rangap* mutations diminish Pros export in the larval central brain NBs.

## 3 DISCUSSION

Pros is a homeodomain transcription factor expressed in NBs and contains both a nuclear localization signal (NLS) and a nuclear export signal (NES; Demidenko et al., 2001; Vaessin et al., 1991). Our data suggest that an intrinsic mechanism maintains Pros shuttling across the nuclear envelope in the larval brain NBs. Mutations in *rangap* disrupt this mechanism and lead to the impaired export of Pros, causing early accumulation of Pros in the nucleus and premature NB cell cycle exit. Our Caspase 3 staining and P35 expression data support the conclusion that apoptosis did not play a role in *rangap* NB missing phenotype.

### 3.1 RanGAP is required for NB maintenance in late larval stage

MARCM analysis shows that with the attenuation of RanGAP, the premature nuclear accumulation of Pros only occurs after 72 hr ALH. At earlier developmental stages, the lack of RanGAP does not appear to be necessary to prevent Pros premature retention in the nucleus. This observation may explain the relatively weak phenotype of NB loss in the second‐instar larvae of both homozygous null alleles.

One of the possible reasons why nuclear Pros is only detected from 72 hr (ALH) onwards might be due to the maternal effect of *rangap*. Since *rangap* null alleles only survive to the second‐instar larval stage (48 hr ALH), zygotic *rangap* must be required after 48 hr (ALH). If it were maternal *rangap* that prevented the retention of nuclear Pros, we would expect to observe the phenotype at the second‐instar larval stage (48 hr ALH). It is possible that other factors might have redundant functions preventing the nuclear retention of Pros. Alternatively, Pros might not shuttle across the nuclear envelope at an earlier developmental stage.

### 3.2 Defective Ran cycle leads to earlier retention of nuclear Pros in the larval NBs

It is interesting to note that mutations in *rangap* shared the same phenotype as the *Rcc1* mutant (Joy et al., 2015). Ran plays a critical role in nucleocytoplasmic transport. Partitions of RanGAP in the cytoplasm and Rcc1 in the nucleus facilitate the Ran cycle and maintain a high RanGTP/RanGDP ratio in the nucleus and a low ratio in the cytoplasm. The low RanGTP/RanGDP ratio in the cytoplasm is likely interrupted by the attenuated RanGAP levels (causing an increase of RanGTP). Similarly, in the absence of Rcc1, the high RanGTP/RanGDP ratio in the nucleus is also compromised (as evidenced by the increase of RanGDP). It is likely that these altered RanGTP/RanGDP ratios lead to a malfunctioned export function, resulting in premature retention of nuclear Pros in the mutant NBs. This conclusion is supported by the genetic evidence that the removal of one copy of Rcc1 in the *rangap^[EP1173]^* background rescues this phenotype.

### 3.3 An intrinsic mechanism maintains Pros shuttling across nuclear envelope

Our Importin experimental data are consistent with our proposal that an intrinsic mechanism maintains Pros shuttling across the nuclear envelope in larval central brain NBs. The fact that Pros contains a NLS and a NES supports this hypothesis. When *importin β *was knocked down in interphase NBs, Pros was largely cortically enriched. It is logical to argue that if Pros is not imported into the nucleus continuously by the nucleocytoplasmic transport system, attenuation of Importin β should not alter Pros distribution. Although we cannot exclude the possibility that Pros forms a complex with Importin β in the cytoplasm to prevent its cortical localization in interphase NBs, the results of our import/export double‐blocking experiment did not support this possibility. The Pros antibody staining signals from *importin β* RNAi knockdown in either *wt* or *rangap* mutant NBs showed very different patterns (cortical vs. barely detectable).

Nup 107, Nup 160, and Nup 98‐96 are the members of the NPC. These three proteins were identified as suppressors in a genetic screen to rescue the G4C2 repeats‐mediated neurotoxicity in *Drosophila* eyes (Freibaum et al., 2015). These three proteins function to facilitate protein export. Attenuation of any one of these proteins in the larval brain NBs recapitulates the *rangap* mutant phenotype. This indicates that impaired Pros export out of the nucleus is able to cause retention of Pros in the nucleus.

It is interesting to note that the transcription factor Ase is present in the nucleus of the NBs, even when Importin β expression is knocked down (Figure 6c,d). This may suggest that different transcription factors use different mechanisms to enter the nucleus. The intrinsic mechanism maintaining import/export of Pros in the NBs seems to be protein‐specific. It is not clear whether other proteins containing both NLS and NES also use this mechanism to shuttle across the nuclear envelope.

### 3.4 Potential end target of the temporal specification mechanism

The intrinsic Pros shuttling mechanism could serve as an effective end target of the temporal series of stereotypical transcription factors that controls the lifespan of the NBs in the central brain. It is conceivable that in the pupal brain, the last transcription factor of the temporal series downregulates RanGAP cytoplasmic levels, which leads to impaired export of Pros. The retention of nuclear Pros then triggers the signal for NB cell cycle exit and termination of NB proliferation potential. A similar mechanism has been observed in the mammalian neurogenesis when, at the very beginning of neuronal terminal differentiation, RanGAP levels were drastically decreased in mammalian primary cortical progenitor cells. This attenuation of RanGAP levels irreversibly triggered the cell cycle exit of progenitor cells (Fujiwara et al., 2016).

**Figure 7:**
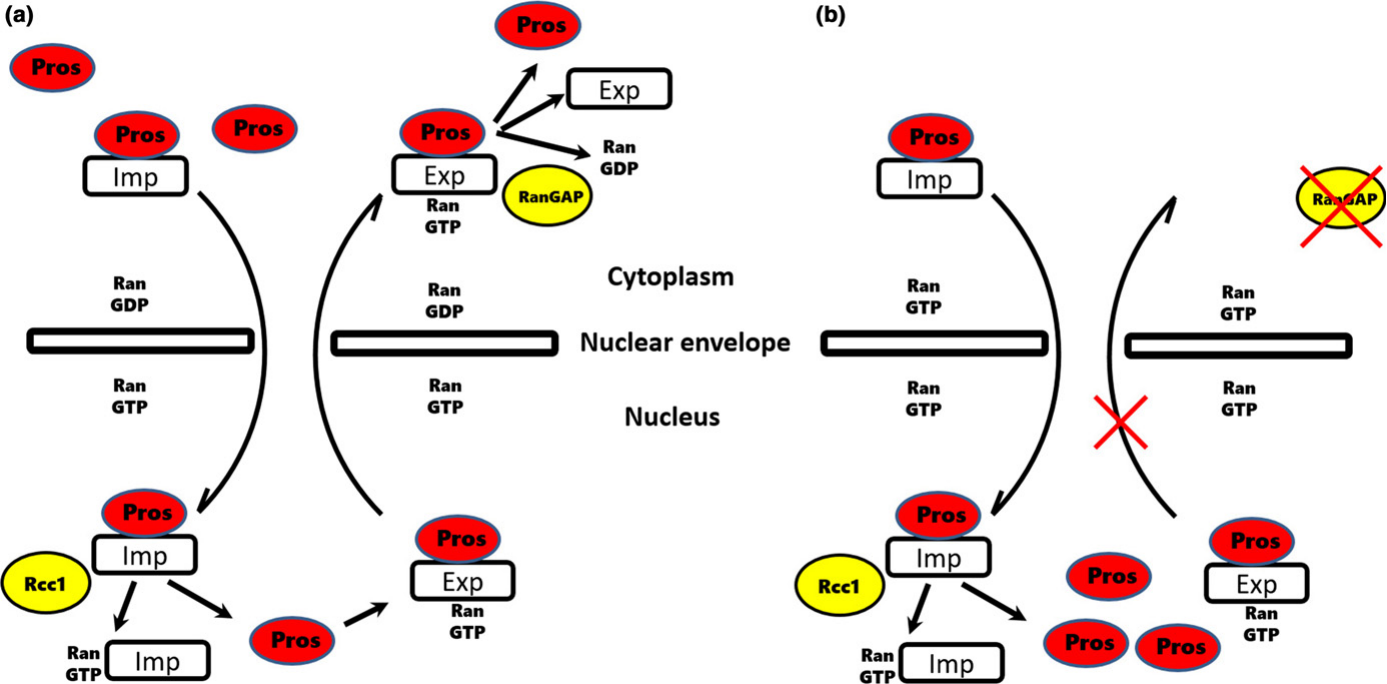
Diagram depicting RanGAP function in the maintenance of Pros shuttling across the nuclear envelope in the larval central brain NBs. This mechanism includes additional components of the nucleocytoplasmic transport system (a). During the process of import, Pros binds to Imp and enters into the nucleus then GTP binds to Imp and releases Pros. During the process of export, Pros binds to GTP and Exportin. In the cytoplasm, GTP will be hydrolyzed into GDP which permits Pros release. It seems RanGTP/RanGDP plays a more important role in Pros export than import. In the absence of RanGAP (b), only the export of Pros is compromised while the import of Pros is not affected in the larval brain NBs. Exp: Exportin; Imp: Importin

In summary, we have identified an intrinsic mechanism that maintains Pros shuttling across the nuclear envelope in the larval brain NBs (Figure 7). This mechanism includes functions of RanGAP and other components of the nucleocytoplasmic transport system (Figure 7a). In the absence of RanGAP, only the export of Pros is compromised in the larval brain NBs (Figure 7b). Under this circumstance, the early retention of Pros in nuclei occurs. This nuclear Pros then leads to a premature NB cell cycle exit. This intrinsic mechanism could be an end target of the temporal series of transcription factors that controls NBs lifespan.

## 4 MATERIALS AND METHODS

### 4.1 *Drosophila* stocks and genetics

The fly stocks and crosses were maintained at 25°C. The following fly stocks were used in this work: *w^1118^*, *P{EP}rangap^EP1173^* (Bloomington, 16995), *P{GawB}Rcc1^NP4610^* (Bloomington, 104713), *rangap^327^* (constructed by P‐element mutagenesis), *rangap* ^9^ (constructed by CRISPR/Cas9), *rangap* RNAi (Tsinghua *Drosophila* center, THU3158), *bj1‐*RNAi (Tsinghua *Drosophila* center, THU3327), *UAS‐p35*, *P{FRT}40A tubP‐Gal80 *(BDSC5192), *{neoFRT}82B/TM6 B*, *{Gal4‐da.G32} UH1/TM3^Sb[1]^*(BDSC27608), *wor‐dicer2; wor‐Gal4*.

To generate the *pUAST‐rangap‐CTAP* construct, the full‐length *rangap* cDNA was amplified with the primers 5′‐GAagatctATGTCCACCTTTAACTTCGC‐3′ and 5′‐GGggtaccTTACGACTCCGCACCCTCCA‐3′ and cloned into a *pUAST –CTAP *vector. This construct was then injected into *w^1118^* embryos using the standard P‐element‐mediated transgenesis protocol. One transgenic line with a third chromosome insertion was obtained and used in this study.

The FLP/FRT system was used to induce mutant clones in the larval brain. Clones were labeled positively (presence of GFP, MARCM; Wu & Luo, 2006). The larvae were heat shocked for 1 hr at 0–4 hr after larvae hatched (ALH). Brains were then dissected and fixed at 48, 72, and 96 hr ALH. The flies used were as follows: Elav‐Gal4 hsFlp UAS‐mCD8::GFP; Tub‐GAL80 FRT40A/Cyo, *rangap^9^* FRT40A/CyoGFP.

### 4.2 Mutant generation

The *rangap^327^* mutant allele was generated by imprecise mobilization of a P‐element insertion *P{EP}rangap^[EP1173]^*using the standard procedure. Sequence analysis revealed the production of a point mutation (Ch2L:19441351 G→T), and this caused an early termination codon. The *rangap^9^* mutant allele was generated by CRISPR/Cas9 method (Yu et al., 2013). The guide‐RNA target sequence was “GGGCGCCAAACTGACAGTCC” and deletion removed five bases (Ch2L:19450932–19450936; GACAG) of the coding region near the N‐terminal and generated an early termination codon.

### 4.3 Immunostaining and microscopy

For brain immunostaining, second‐ and third‐instar larval and pupal brains were dissected in ice‐cold PBS (10 mM NaH2PO4/Na2HPO4, 175 mM NaCl, pH7.4) and fixed for 20 min in PBS with 4% paraformaldehyde (Zhang et al., 2016). We used the following primary antibodies: chicken anti‐GFP (1:2000, ab13970 Abcam); mouse anti‐GFP (1:2000, ab1218 Abcam); rabbit anti‐GFP (1:2000, ab290 Abcam); monoclonal anti‐Prospero (1:5 DSHB); monoclonal anti‐Mira (1:20 F. Matsuzaki); rabbit anti‐Mira (1:500 generated in our laboratory); rabbit anti‐Asense (1:500 C. Yu); Guinea Pig anti‐Dpn (1:500 C. Yu); anti‐E‐cadherin (1:100; DCAD2‐5 DHSB); rabbit anti‐cleaved Caspase 3 (1:100, Asp175 Cell Signaling); and rabbit anti‐phosphohistone 3 (Ser10; 1:500; Millipore, Bedford, MA). Anti‐RanGAP was raised in rabbits against a GST‐RanGAP fusion protein harboring 147 amino acids (aa280 to 426) encoded by the *rangap* gene and used in 1:5000 dilution. Anti‐Rcc1 was raised in Guinea Pigs against a GST‐Rcc1 fusion protein harboring 175 amino acids (aa59 to 233). Secondary antibodies (Alexa Fluor 488, 555, or 633‐conjugated, anti‐rabbit, anti‐mouse, anti‐chicken) were from Molecular Probes (1:2000). TO‐PRO3 (1:5000; Sigma) was used to stain for nuclei.

For 5‐ethynyl‐29‐deoxyuridine (EdU) analysis, late third‐instar larvae were dissected in Schneider’s Drosophila medium, and tissues were incubated for 30 min in 5 mm EdU before fixation. Detection was performed according to the manufacturer’s protocol (C10338, Click‐iT EdU Alexa Fluor 555 Imaging Kit; Life Technologies).

The images were obtained with an Olympus FV1000 confocal microscope and processed using Adobe Photoshop. All statistical data of NB numbers were from quantitation of total central brain NBs in each lobe of the brains.

Animal experiments were conducted in accordance with the Guidelines for the Care and Use of Laboratory Animals of Zhejiang University.

### 4.4 Western blotting

Protein extracts were prepared from larval brains in a lysis buffer (1x RIPA buffer: 50 mM Tris‐HCl pH 8.0, 150 mM NaCl, 1% IGEPAL CA‐630, 0.5% sodium deoxycholate, 0.1% SDS) containing a protease inhibitor cocktail (Roche). The lysates were cleared by centrifugation at 12,000 *g* for 10 min at 4°C. Samples were subjected to SDS‐PAGE and transferred to a polyvinylidene fluoride membrane. Membranes were immunoblotted with the primary antibodies. Rabbit anti‐RanGAP (1:5,000) and mouse anti‐GAPDH (1:1,000; DHSB) were used. Secondary antibodies (HRP, anti‐rabbit, anti‐mouse) were from Abcam and Molecular Probes (1:2000). Blots were treated with the ChemiLucent™ ECL detection reagents (Millipore), and protein bands were visualized using a chemiluminescence imaging system (Clinx Science Instruments, Shanghai).

### 4.5 Statistical analysis

All analyses described below were carried out using the open source softwares GraphPad Prism and ImageJ.

## CONFLICT OF INTEREST

None declared.

## AUTHOR CONTRIBUTIONS

X. Y., Y. X., and D. W. designed the experiments and wrote the manuscript. D. W. and L. W. conducted the RanGAP experiments. P. G. and B. Z. generated the *rangap^9^* mutant allele by CRISPR/Cas9. H. B, H. Z, and F. Z. took part in biochemical and genetic experiments. H. A., W. G., and Y. C. gave many constructive suggestions for the project and valuable inputs for this manuscript.
