# Supplementary figures and images for: RanGAP‐mediated nucleocytoplasmic transport of Prospero regulates neural stem cell lifespan in Drosophila larval central brain
Source: Aging Cell. 2018 Dec 13;18(1):e12854. doi: 10.1111/acel.12854 (PMC6351831; doi:10.1111/acel.12854)

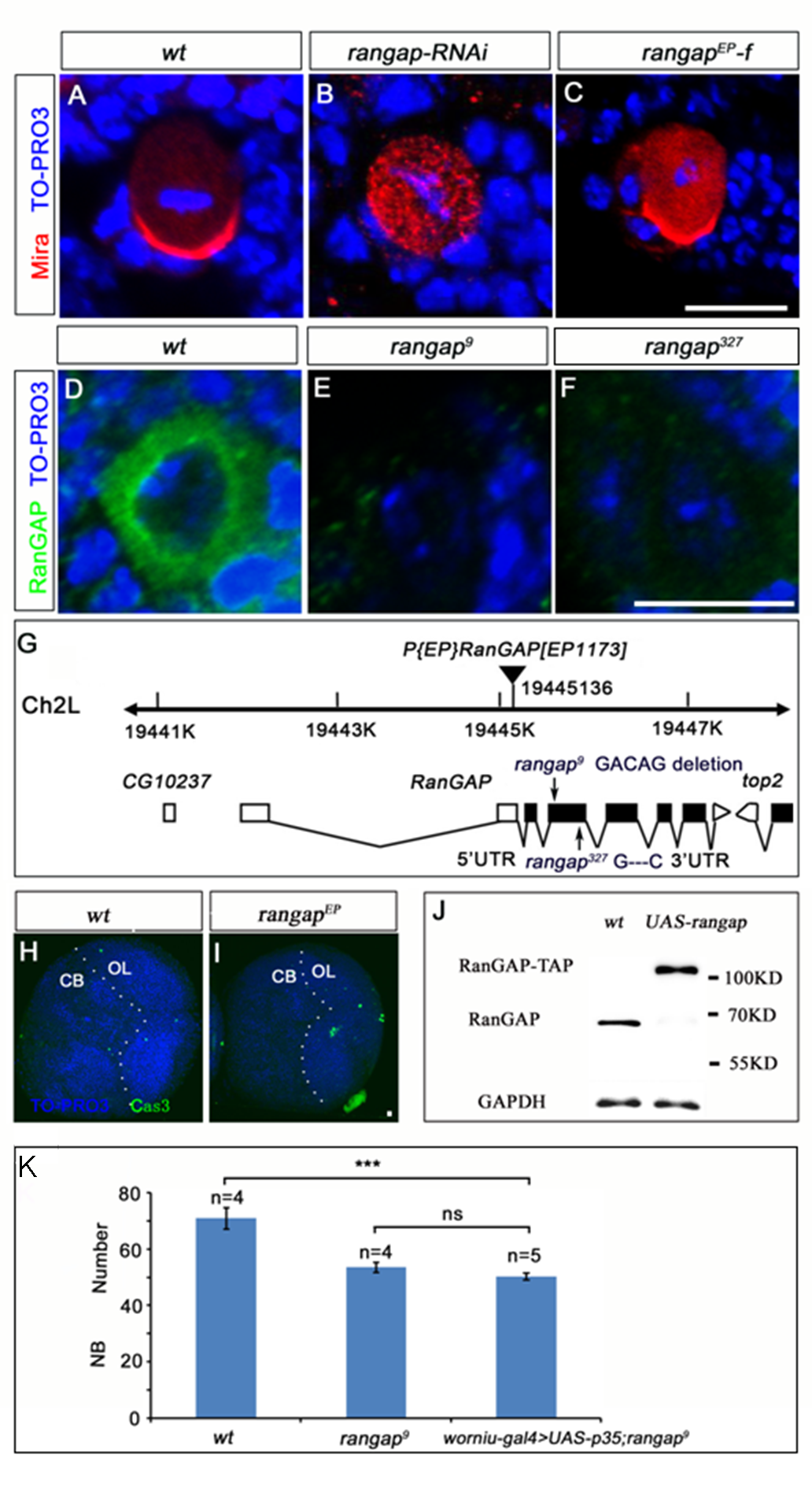

Supplement: Supplementary file 1 [file ACEL-18-e12854-s001.tif]

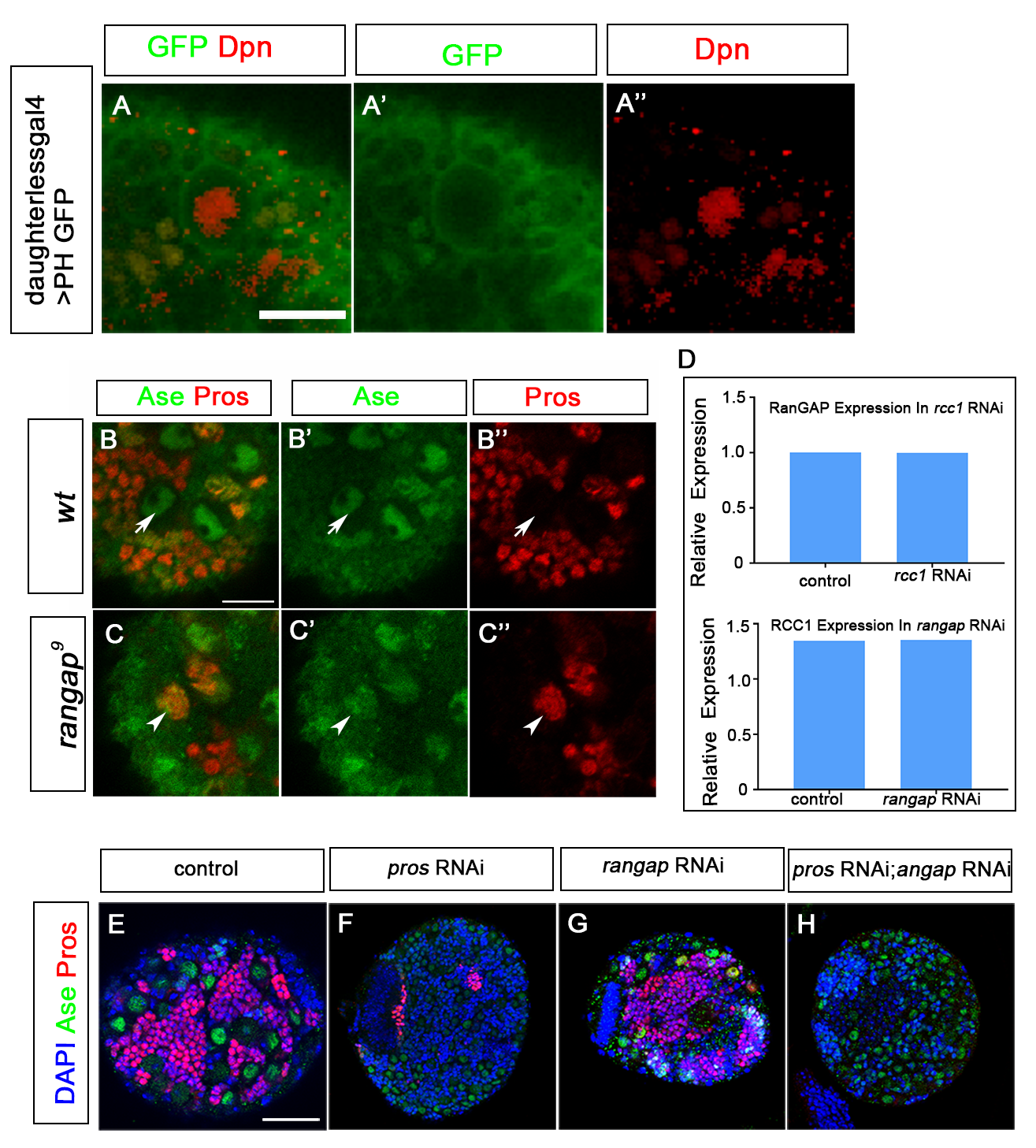

Supplement: Supplementary file 2 [file ACEL-18-e12854-s002.tif]
